# Draft Genome Sequence of *Pseudomonas putida* CA-3, a Bacterium Capable of Styrene Degradation and Medium-Chain-Length Polyhydroxyalkanoate Synthesis

**DOI:** 10.1128/genomeA.01534-17

**Published:** 2018-01-25

**Authors:** Eduardo L. Almeida, Lekha M. Margassery, Niall O’Leary, Alan D. W. Dobson

**Affiliations:** a School of Microbiology, University College Cork, Cork, Ireland; b Environmental Research Institute, University College Cork, Cork, Ireland

## Abstract

*Pseudomonas putida* strain CA-3 is an industrial bioreactor isolate capable of synthesizing biodegradable polyhydroxyalkanoate polymers via the metabolism of styrene and other unrelated carbon sources. The pathways involved are subject to regulation by global cellular processes. The draft genome sequence is 6,177,154 bp long and contains 5,608 predicted coding sequences.

## GENOME ANNOUNCEMENT

Styrene is a solvent used extensively in the polymer-processing industry. It is a toxic compound which is known to have numerous adverse effects on human health (1–4). As a result of this toxicity, there is considerable interest in styrene waste management solutions, including the potential for microbial bioremediation. The bacterium *Pseudomonas putida* CA-3 was isolated from an industrial bioreactor following enrichment on styrene as a sole carbon source (5). In addition to styrene degradation, the organism has also been shown to convert styrene to medium-chain-length polyhydroxyalkanoates (PHA), biodegradable polyesters with physicochemical properties suitable for a range of industrial and medical applications (6). The styrene catabolic pathway in the genus *Pseudomonas* (four major steps) has been described (7). The styrene and PHA pathways have been found to be subject to global regulatory processes in *P. putida* CA-3 (8). However, despite considerable pathway characterization to date, there is still much to be elucidated regarding the overlying cellular mechanisms, including at the genomics level, hence the importance of the draft genome of the strain presented here.

The genomic DNA of the isolate was obtained using the phenol-chloroform-isoamyl alcohol extraction method (9). The sequencing was performed by Macrogen (Seoul, South Korea) using Illumina’s MiSeq paired-end technology. The sequencing generated 3,332,054 reads and 1,002,948,254 bp. The raw data had adapters trimmed using Scythe v.0.994 (see https://github.com/vsbuffalo/scythe) and Sickle v.1.33 programs (10). The reads were then quality filtered and trimmed using FaQCs v.1.35 for a minimum quality value (QV) score of >20, resulting in 3,321,320 reads and 916,019,954 bp, with an approximate coverage of 150-fold (11). The processed reads were assembled *de novo* using SPAdes v.3.10.1, and contigs of <500 bp were removed (12).

The quality of the assembly was assessed using QUAST 4.5, resulting in 92 contigs, 6,177,154 bp, an *N*_50_ of 165,779 bp, and a G+C content of 61.89% (13). The annotation was performed using the NCBI Prokaryotic Genome Annotation Pipeline (PGAP) v.4.3, which predicted 5,608 coding sequences (CDSs), 6 rRNAs, and 70 tRNAs (14).

### Accession number(s).

This whole-genome shotgun project has been deposited in DDBJ/ENA/GenBank under the accession no. PIJT00000000. The version described in this paper is the first version, PIJT01000000.
